# Effects of audio feedback interventions with the Disklavier on the performance of piano students

**DOI:** 10.3389/fpsyg.2025.1568021

**Published:** 2025-05-01

**Authors:** Manfred Nusseck, Friederike Wild, Christoph Sischka, Claudia Spahn

**Affiliations:** ^1^Freiburg Institute for Musicians‘ Medicine, University of Music Freiburg, Medical Center and Faculty of Medicine – University of Freiburg, Freiburg Centre for Music Research and Teaching, Freiburg, Germany; ^2^University of Music Freiburg, Freiburg, Germany

**Keywords:** music performance, feedback intervention, practice support, self-assessment, professional musicians, Disklavier

## Abstract

**Objective:**

At an advanced level, musicians need to consider specific strategies of self-observation and self-evaluation to improve performances. Thus, the use of self-recordings is a suitable method. The purpose of this study was to investigate effects of short feedback interventions with audio playbacks on the performance of piano students.

**Methods:**

For that, 25 advanced piano students were asked to play a prepared piece. The students performed on a Disklavier, a grand piano with digital sensors and electromagnets to record and reproduce MIDI data. In the first intervention group, eight students were listening to a replay of their own performance on the Disklavier. A second intervention group of nine students was additionally able to manipulate this playback in tempo and volume by using a remote control. The third group was the control group with eight students, who did not listen to a playback but read a book instead. After the intervention, the students played the performance a second time and rated the performance compared to the first performance. Furthermore, all performances were acoustically recorded and rated by a group of professional pianists in an online survey.

**Results:**

The results showed significant differences in the self-ratings of the students between the groups in certain musical parameters such as rhythm, agogic, interpretation and musical expression. The intervention groups rated the second performance better in these parameters. The intervention groups did not differ from each other, but did differ from the control group. The external ratings did not show any significant effects.

**Conclusion:**

Overall, listening to one’s own recording seems to have an influence on the process of improving one’s own playing. In addition, the Disklavier showed great potential as a feedback instrument for professional piano students.

## 1 Introduction

Learning to play a musical instrument is an intensive process that involves the acquisition of specialized skills, including instrumental, technical and artistic knowledge. It requires an enormous amount of time, energy, and motivation to practice (Spahn, 2015; Miksza, 2022). In addition to individual lessons with a qualified instrumental teacher, music students spend a lot of time practicing and working in self-study phases (Creech and Gaunt, 2012; Miksza, 2022). Targeted and self-regulating practice strategies are developed over a long period (Hallam et al., 2012; McPherson et al., 2017). Self-regulated learning is an active process based on personal objectives and attentive self-knowledge (Zimmerman, 2002). For that, students need to develop individual self-assessment skills in order to monitor their own progress.

A particular learning strategy is self-evaluation. Musicians always listen to themselves when playing. In terms of self-reflection, self-evaluation helps to make decisions about changes in quality and performance. In this form of self-observation, musicians concentrate on certain aspects of the performance and assess how satisfied they were with the realization of their own goals (Hewitt, 2011). The accuracy of self-evaluation has been compared with ratings provided by experts. It was found that the judgements of the music students were less in agreement with those of the experts (Hewitt, 2011). This could be related to the fact that specific strategies for self-assessment change and become better with more expertise. High school music students showed more accuracy in self-evaluation than junior high students (Hewitt, 2005). The self-evaluation accuracy increases as the students mature.

In particular, in the performance phase of the learning process, musicians use various self-control and self-observation skills that help them to focus their attention on the musical performance (McPherson et al., 2019). A useful procedure in order to assist the self-regulated learning is self-listening to recordings of oneself (Tomita and Barber, 1996). Hearing one’s own performance triggers neurological and cognitive processes, especially in the motor area, which support the learning (Altenmüller and McPherson, 2007). Self-evaluation can increase musical performance when combined with listening to a recording (Hewitt, 2001). For that, self-recordings are considered as valuable feedback (Hewitt, 2001, 2005; Hatfield and Lemyre, 2016). Self-recording has the potential to improve performances by raising awareness (Zimmerman, 2002). The use of this method has increased in recent years due to the ease of making audiovisual self-recordings with a smartphone.

When technical and musical skills are at a high level, self-observation skills are also very well developed and may have reached a plateau. At an advanced stage, a rethinking of practice strategies could be considered to improve performance outcomes. For that, the principles of differential learning of destabilization developed by Schöllhorn might be useful (Schöllhorn, 2019). According to this theory, the learner should perform in a completely different way than usual in order to access other movement and attention potentials that can improve the learning process and the performance outcome.

With the technology of the Disklavier, players are able to listen to their own playing on the same piano from an external observer position. The timing of the key strokes, the speed and dynamics can also be displayed with appropriate software. The Yamaha Disklavier is a grand piano with installed sensor technology to record MIDI without affecting the piano’s key and hammer mechanics. It is also able to replay recordings using electromagnets that accelerate the hammers on the strings according to the MIDI data. Riley et al. (2005) and Riley (2022) showed that using the technology of the Disklavier as a feedback method helped piano players to understand the musical and technical-physiological processes of their own playing. Beyond this, the Disklavier can also be used as a feedback method to train four-handed playing and to practice sight-reading (Sischka et al., 2018; Sischka, 2019).

By using the Disklavier in piano lessons of University students Tomita and Barber (1996) showed that through the recording and playback function, a different insight into the structure of the piece of music, the balance of the voices and the melody line was possible. By reducing the tempo, technical passages could be analyzed more precisely and weak points could be identified.

Moreover, the Disklavier is capable of not only replaying a performance, but also manipulating the playback during the playing. While the Disklavier reruns the recording, the tempo and volume can be changed in real time. For investigating the effect of self-listening of one’s own recording on the self-evaluation, music students were asked to rate their performance after listening to a simple audio recording of that performance or without listening to a replay (Summitt and Fisher, 2016). The ratings were made on scales regarding musical parameters such as intonation, tempo, interpretation and melodic and rhythmic accuracy. The results showed that self-listening had only a small but not significant positive impact on the self-evaluation. The Disklavier provides in contrast to a simple audio recording the possibility to simultaneously hear and change one’s own playing and use this to try out other playing and musical alternatives (Schöllhorn, 2019). It might therefore be possible that effects of musical parameters can be increased by using the manipulation function of the Disklavier.

In previous studies (Tomita and Barber, 1996; Riley et al., 2005; Riley, 2022), the Disklavier was used to analyze piano playing for identifying flaws and structural incongruities. Those were often discussed between player and teacher with the goal to find improvement possibilities. In this study, the Disklavier was used as a playback and interaction device to induce self-evaluation processes without external help and comments.

### 1.1 Aim of the present study

In this study, effects of feedback strategies using the technology of the Disklavier on the performance of self-selected piano pieces were investigated. Our study compared two performances of the same piece by the same player either with or without feedback provided as playback on the Disklavier.

In particular, two variants of feedback through the Disklavier were to be examined and integrated into the study: on the one hand, the widespread variant of listening to one’s own playing via audio recording, and on the other hand, the variant by the Disklavier of manipulating the audio recording of one’s own playing with regard to specific musical parameters.

## 2 Materials and methods

### 2.1 Design

The study was designed as a quasi-randomized controlled experimental study with two intervention groups and a control group. The *first intervention group* had the task of listening to their own recording (audio group: AG). Audio feedback was provided by playing the recording of their performance from the Disklavier. The *second intervention group* listened to the audio recording from the Disklavier, with the task of using a remote control device to edit the playback of the piano piece in real-time (remote control group: RCG). The *control group* (CG) received no audio feedback and spent the same amount of time as the participants in the intervention groups reading a book without reference to the interpretation of the play. The CG and the AG were chosen with reference to the two groups (self-listening and non-listening) of Summitt and Fisher (2016). The RCG was selected as advanced and interactive AG group.

Ahead of the study, all participants received a training of 30 min with the remote control of the Disklavier. The students were able to try out the remote control in a short unit as part of their usual instrumental lessons. This was intended to eliminate possible technical handling difficulties with the remote control as a confounding variable.

The students have made individual appointments for participation. The study time was approximately 30 min for each participant. At the beginning of the study, the students were asked to fill in a questionnaire regarding personal data and an information about the preparation of the piece. The pieces should be new to the players, but already prepared to a good technical level. The students were assigned to *n* = 8 in the audio intervention group (AG), *n* = 9 in the remote control intervention group (RCG) and *n* = 8 in the control group (CG). Before the start of the performances, they were randomly divided into the groups but with taking into account a rather similar gender distribution.

After a short warm-up period on the piano, the students were asked to play the prepared piece once on the Disklavier. The students were recorded while playing the piece on the Disklavier.

During the intervention, the students of the AG were listening to their own playing repeated by the Disklavier. They were listening to the playback twice. The students of the RCG during the intervention were able to manipulate the playback from the Disklavier while listening to their own playing. They did this about two times. Both groups could not see the piano keyboard during the playbacks in order to eliminate the visual feedback as a confounder. The students of the CG were asked to read a book without listening to their playing.

After the intervention phase and the control waiting period, the students had to play the same piece again. Both performances were recorded with the Disklavier as well as with an audio device. All students had only one attempt at each recording. At the end, the students were asked to fill in a questionnaire in order to self-evaluate their playing.

For the comparison of the musical quality of both performances before and after the feedback intervention or waiting period, a self-developed questionnaire to self-evaluate the differences between the performances by the students was used.

In order to validate effects through external assessment on piano playing of the interventions, expert ratings of the piano recordings had taken place. The two performances before and after the intervention were presented acoustically to external raters to provide musical assessments. For that, the external raters were asked to rate each pair of recordings, i.e., the second recording should always be compared to the first. The order of the pairs of first and second recordings was presented randomly. The evaluation was provided as an online survey.

The study was approved by the ethics committee of the University Clinic of Freiburg. The participating students completed a written consent form to participate after reading an informational letter about the study. For the external raters the participation agreement was confirmed on the first page of the online survey.

### 2.2 Participants

Twenty-five music students majoring in piano participated in the study. They were on average 23.1 years old (SD = 3.8 years), 68% (*n* = 17) identifying as female and 32% (*n* = 8) as male. The students reported playing the piano on average 16.6 years (SD = 4.7 years). The mean number of performances per year was 10.4 performances (SD = 7.7). The bachelor of music program was studied by 44% (*n* = 11) and the master of music program by 24% (*n* = 6). Concert exam or master class program were studied by 32% (*n* = 8) of the students. There was no statistically significant difference in the distribution of gender among the study programs.

For the external raters, professional pianists were personally asked to participate. Twelve pianists took part in the survey. Four pianists were female and eight pianists were male. On average, most of the pianist were in the age group of 55 to 59 years (minimum: 40 to 44 years; maximum: 75 to 79 years). All the raters were recognized professional pianists and had experience of judging piano examinations and piano competitions.

### 2.3 The piano pieces

The students were asked to perform a short piano piece of 1–2 min in length. For that, the students were asked to bring a piano piece that they were currently working on or preparing for a public performance. The piece was supposed to be a representative sample of the usual piano literature for concert and competition programs.

They stated that they had been playing the piece they had brought for about 7.1 months (SD = 18.7 months; range 0,25–96 month). When asked if the students had already performed the prepared piece in public, 60% of the students answered in agreement. These students also reported of having performed the piece on average 1.4 (SD = 0.7; range 1–3) times.

### 2.4 Recording devices

For the recordings, the Yamaha Disklavier DS6 Mark IV Pro was used. Parameters of the keystrokes, i.e., the press and release velocity, were recorded in a MIDI data format. In addition, the time between the first press and the last release of the keys was used as duration value of the pieces. The recorded MIDI file was fed into the Disklavier and the piano then repeated the piece. Goebl and Bresin (2003) showed that the Disklavier was precise and reliable as a feedback instrument in performance studies despite small variations in the reproduction of the MIDI recordings.

The device to manipulate the playback of the Disklavier in real-time was developed at the University of Music Freiburg. An acceleration sensor was mounted on a stick of about 20 cm. By tilting the stick forward or backward as well as to the right or to the left, the volume and the tempo were modulated, respectively, in both directions to a range of −50 to +50%.

In another analysis method, the MIDI files of the Disklavier were analyzed. MIDI provides the intensity of the keystroke for each note played. This refers to the volume of the note. In this study, the average loudness of each piece, i.e., the mean intensity values, were calculated. The value has no unit and ranges between 1 and 255. In addition, MIDI records the time point of each key pressed. The time between the first and the last note played was taken to determine the duration of each piece.

The audio was recorded using a Zoom H4n device in a wav format (48 kHz, 24 bit).

### 2.5 Questionnaires

#### 2.5.1 Sociodemographic and study-related data

Sociodemographic data such as age and gender were collected from all participants. The age of the external raters was determined using age groups with 5-year intervals. In addition, the students were asked about their degree program, how long they had been playing their instrument and the average number of performances per year. Regarding the piece, the students were also asked to report how long they had been practicing the piece and if they had already performed it in public.

#### 2.5.2 Questionnaire for music performance evaluation

The questionnaire for music performance evaluation had been self-developed for this study. The questions were designed to compare the second performance with the first performance. For this purpose, a seven-point Likert scale was used, ranging from 1 = “much worse” to 7 = “much better” and with 4 = “same.” The questionnaire was completed by the students as self-rating and by the external raters as external rating.

The questionnaire contained two parts. In the first part, two questions were asked relating to the general impression and the overall musical impression of the performances.

The second part contained 14 musical items, which were used to judge the performances. They were chosen according to Poli (2004), who describes musical expression by certain parameters, the so-called expressive performance parameters. The main parameters among those are timing, tempo, dynamics and articulation, but also pedalization and timbre. These six parameters make part of the questionnaire. The parameter tempo was completed by the parameter rhythm. Instead of timing, the parameter agogic was used. This parameter describes small modifications of tempo, which are not noted in the score and provides individual dynamic expression. In addition to these parameters, other parameters related to musical expression were included considering the possible outcome of the intervention.

Following Nusseck and Wanderley (2009), musical flow describes the fluency of the performance in terms of soft to hard and flowing to halting. Technical confidence is a parameter that takes into account technical aspects of the performance. Evaluating the amount of errors during the performances, the parameter faultlessness of the performance was added. The parameter phrasing refers to the shaping of notes within a musical phrase in terms of loudness, rhythm, articulation, and pausing. Since the volume and intensity can be manipulated using the remote control, the range between loud and soft parameter has been added. Finally, two parameters considering the individual musical intention were included. These are the parameters interpretation and expressivity as well as stylistic differentiation. The parameters used in this study represent the perceptible areas of musical expression in the performances.

### 2.6 Statistics

The analyses of questionnaire items from the self-assessment and the expert questionnaires were performed using SPSS 29 (SPSS Inc., Armonk/NY, USA). Nonparametric distribution tests were calculated using cross-tabulations and determined using Pearson’s Chi^2^. For parametric variables, means were described by the standard deviation (SD) of the mean. For comparative analyses, t-tests and multivariable analyses of variance (ANOVA) were used. On significance, Post-Hoc analyses were performed using the Tukey-HSD correction. The level of significance was set to *p* = 0.05. Estimates of the effect sizes are represented by the partial Eta^2^ (pη^2^) value provided by SPSS.

## 3 Results

### 3.1 Students’ ratings

**Figure 1:**
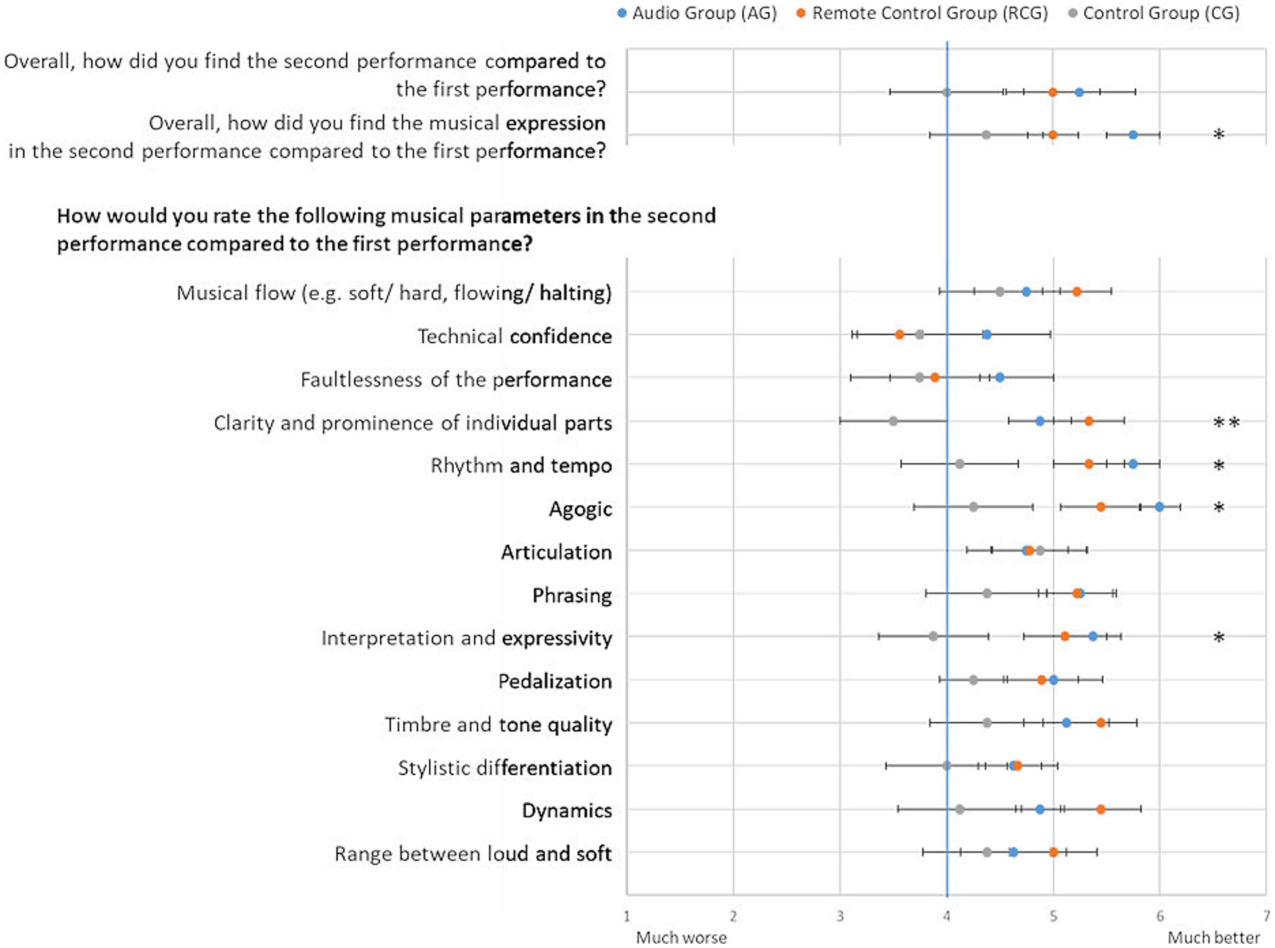
Mean values of the students’ ratings for each parameter by groups (error bar: standard error of the mean; blue line: middle of the scale representing no difference between the first and the second performance; *p < 0.05; **p < 0.01).

The mean values of the students’ ratings, divided among the different groups, are shown in Figure 1. The data and the statistics can be found as tables in the Supplementary material. For the first two questions of general and musical impression, the students in the audio and the remote control groups rated the second performance better than the first performance. The control group rated both performances in these parameters as rather similar. However, there was a significant difference between the groups only for the musical impression (*F*(2,23) = 3.57; *p* = 0.045; pη^2^ = 0.25). The mean value of the audio group was significantly higher than of the other groups (Post-Hoc: *p* = 0.036).

Concerning the musical parameter “clarity and prominence of individual parts,” there was a significant effect between the groups (F(2,23) = 6.19, *p* = 0.007; pη^2^ = 0.36). Students in the audio and remote control group rated the second performance significantly (Post-Hoc: *p* < 0.01) better than the first performance, while the control group rated both performances rather similar.

Similar results were found for the parameters rhythm and tempo (F(2,23) = 4.45, *p* = 0.024; pη^2^ = 0.29), agogic (F(2,23) = 4.75, *p* = 0.019; pη^2^ = 0.30) and interpretation and expressivity (F(2,23) = 3.85, *p* = 0.037; pη^2^ = 0.26). Each time, the students in the intervention group rated the second performance better than the first and the control group had rather similar ratings for both performances. However, there was no significant difference between the audio group and the remote control group.

### 3.2 External ratings

**Figure 2:**
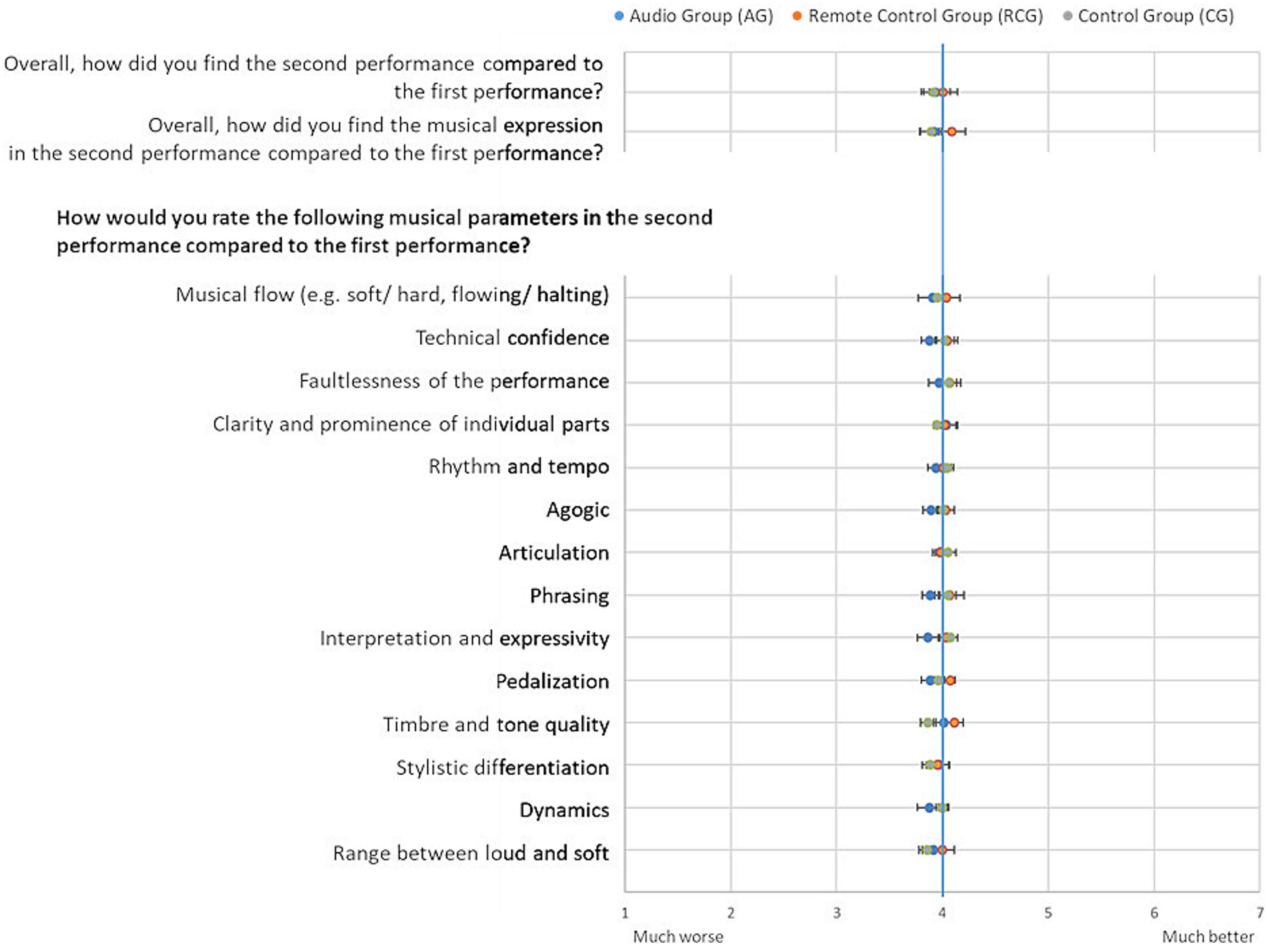
Mean values of the external ratings for each parameter by groups (error bar: standard error of the mean; blue line: middle of the scale representing no difference between the first and the second performance).

In Figure 2 the mean values of the external ratings split by the three groups are shown for each musical parameter. Tables with all data and statistics are in the Supplementary material. The average rating of all musical parameters by all experts was 4.15 (SD = 0.38). This indicates for a similar rating of both performances with a very small variance. There were no significant differences in the ratings of the first and the second performance for any of the musical parameters.

### 3.3 MIDI analysis

The MIDI files of the Disklavier were analyzed for the mean key press intensity (i.e., loundness) and the total duration of the played pieces. The mean intensity value across all players in the first performance was 55.99 (SD = 8.16) and in the second performance 55.50 (SD = 8.11). The mean durations of the pieces in the first performance was 92.1 s (SD = 26.17 s) and in the second performance 92.03 s (SD = 26.60 s).

Since the players performed the pieces with different tempo and intensities at all, the differences between the two performances were calculated. The mean intensity difference was 0.49 (SD = 1.67). The value showed no significant differences between the groups. The mean duration difference was 0.06 s (SD = 2.6 s). The audio group and the remote control group played the second performance slightly faster than the first performance (AG: 0.74 s, SD = 1.12 s; RCG: 0.77 s, SD = 2.82 s). The control group played the second performance a bit slower than the first performance (−1.39 s, SD = 3.03 s). However, the difference between the groups was not significant (*F*(2,22) = 2.031, *p* = 0.155; pη^2^ = 0.16).

## 4 Discussion

In this study, the effects of feedback methods on the performance of piano pieces were investigated. One group of pianists listened to an audio playback of their performance on a Disklavier and a second group was listening to and manipulating their own playback in real-time on a Disklavier. After the second performance, the pianists rated the performances. The results were compared with a control group of pianists reading a book instead.

The results showed that the piano students of both intervention groups (AG and RCG) rated their second performance better than the first. This was especially found for the overall musical expression. This showed that a feedback of one’s own performance could have an effect on the self-perceived musical interpretation and led to an improvement in the second performance. This finding confirms with other studies on the positive effects of listening to one’s own playing (Tomita and Barber, 1996; Altenmüller and McPherson, 2007; Volioti and Williamon, 2017).

The performance changes and the self-evaluation ratings, however, were reported similarly by both intervention groups. There were no significant differences between the two interventions. The theoretical approach that the manipulation of the playback may cause a larger effect as it was proposed by the differential learning theory (Schöllhorn, 2019) could therefore not be confirmed by the results. An explanation for this could be that the students were not yet sufficiently familiar with the handling of the remote control despite previous instruction and therefore focused more on the technical manual handling than on the additionally generated change in the sound result. With regard to the findings of Summitt and Fisher (2016) who did not find significant differences in the self-evaluation between both groups of self-listeners (AG) and non-listeners (CG), it could be expected that the audio playback of the Disklavier would not have any significant effects either. In our study, however, the ratings of the students listening to the Disklavier playback showed differences to the CG. For the above-mentioned musical parameters, they rated the second performance better than the first performance indicating for a learning effect by listening to the replay on the Disklavier. Since the condition of the audio feedback differed between Summitt and Fisher and our study—audio device versus Disklavier—it seems interesting to ask whether the effect measured in our study is due to the use of the Disklavier. Since listening to an audio recording was not measured as a direct comparison condition in our study, this question cannot be answered reliably from the available data. It can only be hypothesized that, in line with the findings of Tomita and Barber (1996) that the use of the Disklavier provides specific insights into the performance, the Disklavier as an advanced MIDI playback device on a real piano could provide more information than just a simple audio playback. A potential difference between a playback on the Disklavier or a simple audio device should be investigated in further research.

Furthermore, both intervention groups rated the clarity and prominence of individual parts, the rhythm and tempo, the agogic as well as the interpretation and expressivity of the second performance significantly better than the first performance. This shows that the playback in general was able to let the players focus on these specific musical parameters. The students were able to use this information to include it in the second performance. The self-ratings showed their success.

The result that other musical parameters seemed mainly unaffected by the playback in both intervention groups could be explained by the rather high performing level of the students and the fact that the pieces were expected to be well prepared before the study. At a high performance level, playing is already so advanced that certain technical skills are already highly developed. When a piece reaches a certain level of practicing, basic performance cues such as technical features (e.g., fingerings) and interpretative clues such as phrasings, dynamics, tempo and timbre are mostly finalized (Chaffin and Logan, 2006). These are components of the interpretation of the generative rules of music that group harmonic structures and convey communicative accents (Davidson and Broughton, 2022). When performing, musicians focus on expressive cues that emphasize the musical intention and expression (Chaffin and Logan, 2006). Thus, these parameters seem to be influenced and changed by feedback and analysis. They also differ between performances (Davidson, 2011). With the intervention in this study, the students used the playback as information source to attend to the expressive parameters and changed them in the second performance to self-perceived more successful outcome.

The analysis of the MIDI files showed that there was no significant difference in the length of the pieces before and after the intervention between the groups. There was only a slight tendency that the intervention groups played their pieces the second time a bit faster where the CG played the second performance marginally slower. It is difficult to draw conclusions that this might be an effect of the playback. More research is needed to substantiate this approach.

To investigate if the performance differences can be detected by external listeners, both performances of each player were rated by professional pianists. The assessment of the musical parameters showed no differences at all between the three groups. The professional pianist’s ratings were entirely in the middle range of the scale and so the performances were considered rather equal. Any kind of changes made by the student pianists in the second performance seemed not to be recognizable by the external raters and did not result in different judgments. The raters, who were listening to the recordings before and after the intervention in random order for the evaluation, were unable to assign both recordings in the correct order.

There are at least two possible explanations for this result. One is that it was very difficult for the experts to hear differences between the performances. This could be because the pieces were at a fairly high performance level or because the changes of the students were very subtle and therefore difficult to recognize. The other is that the experts did find differences but could not transfer them into the provided rating scale. As the raters were pianists who are experienced in jury and examination situations, the purely quantitative comparative assessment on scales may have left too little room for the qualitative competence of the raters. For example, assessments in the areas of musical expression and interpretation, which the test subjects consider to be effects, are usually parameters that are differentiated more in qualitative assessments. Also, in contrast to the test subjects, the raters had a different relationship to the audio sample than the test subjects, technically due to the online sound recordings. Overall, a number of methodological questions arise with regard to the external ratings.

### 4.1 Limitations

One particular limitation of this study is that the sample size was rather small. A larger sample would be required for a better understanding and confirmation of the effects. It would also be interesting to include samples of students varying in age and experience. However, the study is technically quite complex and can only be carried out with sufficiently trained test personnel.

Another constrain could be that the intervention of listening to and manipulating a playback might have been too short to trigger effects. It should be examined as part of a longer intervention in order to enable learning processes, especially with more training with the remote control function. A possibility could be to integrate the intervention in regular piano classes. Further research is needed to investigate for long-term learning effects.

Furthermore, the external rating was conducted using quantitative assessment scales that may not have captured the subtle details of the performance differences. The rationale behind this was to use the same scales as the self-evaluation in order to be able to make comparisons. However, it could be that the experts paid attention to other judgement criteria than those indicated by the given musical parameters. It is necessary to carry out further research, which could also include qualitative statements of the experts.

Finally, the analysis of the MIDI file was rather limited. It was not possible to analyze for micro differences in tempo and agogic at specific time points in each piece. The analysis used in this study was rather rudimentary and focused only on simple parameters such as intensity and duration. A more detailed analysis should be carried out in further studies.

## 5 Conclusion

To summarize, it can be said that the short feedback of listening to one’s own performance with the Disklavier had a positive effect on the self-evaluation. This feedback method was used to integrate changes in expressive aspects into the repeated playing of the same piece. The potential of the Disklavier as a feedback instrument seemed to be evident, even though the self-reported progresses were not recognizable for external listeners. The feedback strategy of self-listening seems to enable a better internal understanding of one’s own playing in order to utilize this for improving performances. The method of listening to one’s own playing should be integrated into teaching units as a feedback approach especially for advanced players. Future studies should investigate the comparison between simple audio feedback and audio feedback through the Disklavier in order to further explore the multiple potentials of the Disklavier for learning strategies and its role as a tool in music lessons.

## Data Availability

The raw data supporting the conclusions of this article will be made available by the authors, without undue reservation.
