# Supplementary material for: Effects of audio feedback interventions with the Disklavier on the performance of piano students
Source: Front Psychol. 2025 May 1;16:1568021. doi: 10.3389/fpsyg.2025.1568021 (PMC12078165; doi:10.3389/fpsyg.2025.1568021)
Supplement: Supplementary file 1 [file Data_Sheet_1.pdf]

Supplemental material of the article:

Effects of audio feedback interventions with the Disklavier on the performance of piano students

Manfred Nusseck, Friederike Wild, Christoph Sischka, Claudia Spahn

Descriptions of the questions:

	TOPIC OF THE QUESTION
**OVERALL A**	Overall, how did you find the second performance compared to the first performance?
**OVERALL B**	Overall, how did you find the musical expression in the second performance compared to the first performance?
**QUESTION 1**	Musical flow (e.g. soft/ hard, flowing/ halting)
**QUESTION 2**	Technical confidence
**QUESTION 3**	Faultlessness of the performance
**QUESTION 4**	Clarity and prominence of individual parts
**QUESTION 5**	Rhythm and tempo
**QUESTION 6**	Agogic
**QUESTION 7**	Articulation
**QUESTION 8**	Phrasing
**QUESTION 9**	Interpretation and expressivity
**QUESTION 10**	Pedalization
**QUESTION 11**	Timbre and tone quality
**QUESTION 12**	Stylistic differentiation
**QUESTION 13**	Dynamics
**QUESTION 14**	Range between loud and soft

Self-ratings of the music students:

Mean and standard deviation (SD) of all questions

QUESTION	AUDIO GROUP (AG)		CONTROL GROUP (CG)		REMOTE CONTROL GROUP (RCG)	
	Mean	*SD*	*Mean*	*SD*	*Mean*	*SD*
**OVERALL A**	5.25	1.48	4.00	1.51	5.00	1.32
**OVERALL B**	5.75	0.70	4.38	1.50	5.00	0.70
**QUESTION 1**	4.75	1.38	4.50	1.60	5.22	0.97

**QUESTION 2**	4.38	1.68	3.75	1.66	3.56	1.33
**QUESTION 3**	4.50	1.41	3.75	1.83	3.89	1.26
**QUESTION 4**	4.88	0.83	3.50	1.41	5.33	1.00
**QUESTION 5**	5.75	0.70	4.13	1.55	5.33	1.00
**QUESTION 6**	6.00	0.53	4.25	1.58	5.44	1.13
**QUESTION 7**	4.75	1.58	4.88	1.24	4.78	1.09
**QUESTION 8**	5.25	0.88	4.38	1.59	5.22	1.09
**QUESTION 9**	5.38	0.74	3.88	1.45	5.11	1.16
**QUESTION 10**	5.00	1.30	4.25	0.88	4.89	1.05
**QUESTION 11**	5.13	1.12	4.38	1.50	5.44	1.01
**QUESTION 12**	4.63	0.74	4.00	1.60	4.67	1.11
**QUESTION 13**	4.88	0.64	4.13	1.64	5.44	1.13
**QUESTION 14**	4.63	1.40	4.38	1.68	5.00	1.22

Statistics between the three groups (F-value, p-value and partial Eta²)

	F-VALUE	***P-VALUE***	***PART. ETA²***
**OVERALL A**	1.707	.205	.134
**OVERALL B**	3.569	**.045**	.245
**QUESTION 1**	0.649	.532	.056
**QUESTION 2**	0.624	.545	.054
**QUESTION 3**	0.561	.578	.049
**QUESTION 4**	6.191	**.007**	.360
**QUESTION 5**	4.445	**.024**	.288
**QUESTION 6**	4.751	**.019**	.302
**QUESTION 7**	0.020	.980	.002
**QUESTION 8**	1.346	.281	.109
**QUESTION 9**	3.845	**.037**	.259
**QUESTION 10**	1.105	.349	.091
**QUESTION 11**	1.677	.210	.132
**QUESTION 12**	0.789	.467	.067
**QUESTION 13**	2.542	.102	.188
**QUESTION 14**	0.406	.671	.036

External ratings of the experts:

Mean and standard deviation (SD) of all questions

QUESTION	AUDIO GROUP (AG)		CONTROL GROUP (CG)		REMOTE CONTROL GROUP (RCG)	
	Mean	*SD*	*Mean*	*SD*	*Mean*	*SD*
**OVERALL A**	3.93	0.47	3.91	0.32	4.00	0.45
**OVERALL B**	3.92	0.48	3.89	0.34	4.09	0.43
**QUESTION 1**	3.90	0.48	3.95	0.25	4.04	0.43
**QUESTION 2**	3.87	0.24	4.02	0.31	4.04	0.34
**QUESTION 3**	3.96	0.34	4.06	0.38	4.06	0.23
**QUESTION 4**	4.03	0.38	3.94	0.12	4.02	0.34
**QUESTION 5**	3.93	0.25	4.04	0.20	4.00	0.26
**QUESTION 6**	3.89	0.26	4.00	0.17	4.02	0.28
**QUESTION 7**	3.97	0.22	4.04	0.28	3.98	0.17
**QUESTION 8**	3.88	0.26	4.05	0.25	4.06	0.46
**QUESTION 9**	3.86	0.33	4.07	0.25	4.03	0.21
**QUESTION 10**	3.88	0.28	3.95	0.13	4.07	0.17
**QUESTION 11**	4.01	0.35	3.86	0.25	4.11	0.30
**QUESTION 12**	3.96	0.34	3.88	0.25	3.95	0.36
**QUESTION 13**	3.87	0.38	4.00	0.15	4.00	0.20
**QUESTION 14**	3.91	0.36	3.85	0.26	3.99	0.38

Statistics between the three groups (F-value, p-value and partial Eta²)

	F-VALUE	*P-VALUE*	*PART. ETA²*
**OVERALL A**	0.159	.854	.010
**OVERALL B**	0.748	.481	.043
**QUESTION 1**	0.333	.719	.020
**QUESTION 2**	1.072	.354	.061
**QUESTION 3**	0.340	.714	.020
**QUESTION 4**	0.282	.756	.017
**QUESTION 5**	0.560	.576	.033
**QUESTION 6**	0.974	.388	.056
**QUESTION 7**	0.378	.688	.022
**QUESTION 8**	1.038	.366	.059
**QUESTION 9**	1.971	.155	.107

**QUESTION 10**	2.558	.093	.134
**QUESTION 11**	1.967	.156	.107
**QUESTION 12**	0.220	.803	.013
**QUESTION 13**	0.817	.450	.047
**QUESTION 14**	0.535	.591	.031
